# The Complex Dynamics of Caregiving for ADRD Patients in Uganda: Selection Factors, Challenges, and Benefits

**DOI:** 10.21203/rs.3.rs-10625978/v1

**Published:** 2026-09-03

**Authors:** Joy Louise Gumikiriza- Onoria, Mark Kaddu- Mukasa, Kamada Lwere, Dennis Buwembo, Rita Nassanga, Noeline Nakasujja

**Affiliations:** Makerere University; Makerere University; Makerere University; Makerere University; Makerere University; Makerere University

**Keywords:** Alzheimer’s disease, caregiving, dementia, Uganda, qualitative study, caregiver challenges, caregiver training

## Abstract

**Background:**

Family caregivers provide most of the care for people living with Alzheimer’s disease and related dementias (ADRD), often without preparation or formal support. While caregiver burden is well documented, little is known about how caregivers are selected within families, the challenges they face, and the personal meaning they derive from caregiving in Uganda. This study explored caregiver selection, caregiving challenges, and perceived benefits in a low-resource setting.

**Methods:**

This qualitative study was conducted in Wakiso District, Uganda, using in-depth interviews with 14 family caregivers purposively selected from a previous caregiver cohort. Interviews were conducted in Luganda, transcribed verbatim, translated into English, and analysed using thematic analysis. The Mental Models Framework guided the interpretation of caregivers’ experiences.

**Results:**

Three interrelated themes emerged: relational foundations of caregiving, multidimensional struggles of caregiving, and benefits of caregiving. Caregiver selection was shaped by longstanding family relationships, emotional closeness, a sense of responsibility, and, in some cases, the preferences of the person living with ADRD. Caregivers described overlapping emotional, physical, financial, and social challenges that frequently required substantial personal sacrifices, including leaving employment or education to provide care. At the same time, many reported positive experiences, including increased knowledge of dementia, greater empathy, stronger relationships with healthcare providers and community members, and a sense of fulfilment through giving back to relatives who had previously cared for them.

**Conclusion:**

Caregiving for people living with ADRD in Uganda is both demanding and deeply rooted in family relationships and cultural expectations. While caregivers face considerable pressures, they also find meaning and personal growth through caregiving. These findings underscore the urgent need for culturally tailored interventions that combine education, psychosocial support, practical skills training, and community-based resources to strengthen caregiver wellbeing and improve dementia care in sub-Saharan Africa.

## Introduction

Alzheimer’s disease and related dementias (ADRD) are progressive neurodegenerative disorders that affect millions of people worldwide and are among the leading causes of disability and dependency in older adults [1,2]. As the disease progresses, individuals gradually lose their ability to perform everyday activities, making long-term care increasingly necessary. Across most settings, this responsibility falls primarily to informal family caregivers who provide assistance with personal care, medication management, behavioural symptoms, decision-making, and emotional support [3–5]. Although family caregivers are central to dementia care, they frequently assume this role with little preparation or formal training [6,7]

Providing care for a person living with ADRD is physically, emotionally, socially, and financially demanding. Caregivers commonly experience psychological distress, anxiety, depression, social isolation, financial strain, and reduced quality of life while responding to the progressive cognitive, functional, and behavioural changes associated with dementia [7–9]. Beyond the practical demands of caregiving, families often face uncertainty regarding disease progression, increasing dependence, and limited access to specialised dementia services, particularly in low-resource settings [10,11]. However, caregiving experiences are shaped not only by the clinical manifestations of dementia but also by broader social and cultural influences, including family relationships, gender roles, cultural expectations, and the availability of formal support systems [2,12].

Although the global literature has substantially advanced our understanding of caregiver burden, resilience, and support needs, much of this evidence originates from high-income countries where health systems and social care structures differ considerably from those in sub-Saharan Africa [6,7]. In Uganda, dementia care is delivered almost entirely by family members, yet little is known about how caregivers are selected, the factors that shape caregiving responsibilities, or how caregivers perceive both the challenges and the potential benefits of providing long-term care. Understanding these experiences is essential for designing culturally responsive interventions that strengthen caregiver capacity while improving the quality of care for persons living with dementia. While previous studies have largely focused on caregiver burden and mental health outcomes, considerably less attention has been given to how caregivers are selected within families and how they make sense of the caregiving role in low-resource settings.

This study therefore explored the experiences of family caregivers of persons living with ADRD in Wakiso District, Uganda. Specifically, it examined the relational factors influencing caregiver selection, the multidimensional challenges encountered during caregiving, and the perceived benefits associated with the caregiving role. By providing a contextual understanding of caregiving within a low-resource setting, this study aims to inform the development of culturally appropriate caregiver support interventions and dementia care policies.

## Methods

### Study design

This qualitative study employed an interpretive thematic analysis to explore the experiences of family caregivers of persons living with Alzheimer’s disease and related dementias (ADRD) in Wakiso District, Uganda. The study was guided by the Mental Models Framework [13], which provides a structured approach for understanding how individuals develop internal explanations of unfamiliar situations and use these explanations to interpret their experiences and guide decision-making. Within this study, the framework informed exploration of how caregivers understood caregiver selection, interpreted the challenges they encountered, and perceived the benefits associated with providing care.

### Study setting

The study was conducted between February and July 2023 in Wakiso District, Central Uganda. Wakiso surrounds Kampala, Uganda’s capital city, and forms part of the Greater Kampala Metropolitan Area. The district comprises urban, peri-urban, and rural communities and is home to nearly two million people, including a rapidly growing older adult population [14]. Two sub-counties, Nansana and Busukuma, were purposively selected to capture caregiving experiences across both urban and rural settings.

### Participants and recruitment

Participants were family caregivers aged 18 years and older who were providing care to persons living with ADRD. Caregivers were purposively selected from a previous cohort study investigating the burden of Alzheimer’s disease in Wakiso District. Sampling sought variation in age, sex, and relationship to the care recipient to capture diverse caregiving experiences. One caregiver was recruited from each identified household, and recruitment continued until thematic saturation was achieved. Fourteen caregivers participated in the study.

### Data collection

Data were collected through in-depth interviews conducted by the first author (JLGO) and two trained research assistants with mental health research experience. Interviews lasted approximately 90–120 minutes and were conducted in Luganda, the participants’ preferred language.

The interviews formed part of the first author’s broader PhD research examining how family caregivers provide and manage care for persons living with ADRD. A semi-structured interview guide was developed in consultation with senior investigators and with reference to the Mental Models Framework. The guide used broad, open-ended questions about caregivers’ everyday experiences, the care they provided, how they approached and adapted caregiving tasks, and the support available to them. It was pilot-tested in a neighbouring community that was not included in the study. Interviews began with the invitation, “Tell me about your experience caring for your patient.” Follow-up questions and probes were used flexibly in response to each participant’s account. Interviews were audio-recorded with participants’ permission, and field notes were maintained throughout the interviews to capture contextual observations. The English-language interview guide is provided as Supplementary File 1.

### Data management and analysis

Interviews were transcribed verbatim in Luganda before being translated into English by trained research assistants. The first author reviewed all transcripts against the original audio recordings to ensure accuracy and completeness, while an independent senior researcher reviewed the transcripts to enhance methodological rigour.

Data were analysed using thematic analysis. The first author repeatedly read the transcripts to achieve familiarisation before manually generating inductive codes. Related codes were grouped into categories and subsequently refined into broader themes through iterative discussions among the research team. Throughout the analytical process, the Mental Models Framework provided an interpretive lens for understanding how caregivers constructed meaning around caregiver selection, caregiving responsibilities, and their lived experiences. Attention was given to identifying similarities, differences, and divergent experiences across participants to ensure that the final themes reflected the breadth and complexity of caregiving experiences.

### Trustworthiness

Trustworthiness was enhanced through several strategies. Credibility was strengthened through prolonged engagement with participants, verbatim transcription, and verification of transcripts against the original audio recordings. Purposive sampling ensured representation of diverse caregiving experiences, while data collection continued until thematic saturation was achieved.

Dependability was enhanced through the use of a pilot-tested interview guide and consistent interviewing procedures. Confirmability was promoted through collaborative discussions among the research team during coding and theme development, allowing interpretations to be challenged and refined. Transferability was supported by providing detailed descriptions of the study setting, participant characteristics, and research procedures to enable readers to assess the applicability of the findings to similar settings.

### Ethical considerations

Ethical approval was obtained from the Makerere University School of Health Sciences Research and Ethics Committee and the Uganda National Council for Science and Technology (Approval No. HS2909ES). All participants were aged 18 years or older and provided written informed consent before enrolment. Participation was voluntary, and participants could decline participation or withdraw from the study at any time without consequence. Confidentiality was protected through the use of unique participant identification codes and secure storage of study data. All procedures involving human participants were conducted in accordance with relevant institutional and national guidelines and regulations and the Declaration of Helsinki.

## Results

A total of 14 in-depth interviews were conducted with family caregivers of persons living with Alzheimer’s disease and related dementias (ADRD). Seven participants were recruited from Nansana Town Council and seven from Busukuma Sub-county. Participants ranged in age from 20 to 56 years and included nine women and five men. Their relationships with care recipients included spouses, adult children, grandchildren, siblings, in-laws, and paid caregivers (Table 1).

Analysis of the interviews generated three interrelated themes describing caregivers’ experiences: (1) Relational foundations of caregiving, (2) Multidimensional struggles of caregiving, and (3) Benefits of caregiving (Table 2). Together, these themes illustrate how caregivers entered the caregiving role, the challenges they encountered while providing care, and the personal meaning they derived from caring for a family member living with ADRD.

### Theme 1: Relational foundations of caregiving

Caregiver selection was deeply rooted in existing family relationships rather than formal decision-making processes. Participants consistently described assuming caregiving responsibilities because of longstanding family ties, emotional attachment, a sense of familial responsibility, and the expressed wishes of the person living with dementia. Three interconnected factors shaped caregiver selection: familial relationships and responsibility, emotional connections, and the preferences of the person living with ADRD.

### Familial relationships and responsibility

Participants described caregiving as a natural extension of longstanding family relationships. Close kinship ties, shared life experiences, and previous care received from the older person created a strong sense of responsibility to assume the caregiving role when dementia developed. Rather than being formally assigned, caregiving was commonly viewed as an expected family responsibility.

One participant explained:

“It was a unanimous selection from my siblings; they felt I was close to Mum and she would understand me better.”

(Female caregiver)

Another reflected on how earlier family support influenced her decision to become a caregiver:

“I grew up with my grandmother. She supported me through school and was my mother figure. When she fell sick, I stayed to take care of her.”

(Female caregiver)

These accounts illustrate how caregiving responsibilities often evolved from longstanding family relationships and a shared history of care and support.

### Emotional connections

Beyond family relationships, emotional attachment strongly influenced who assumed caregiving responsibilities. Participants described relationships characterised by affection, companionship, and mutual trust, which strengthened their commitment to continue providing care despite the increasing demands associated with dementia.

One caregiver shared:

“The patient loves me so much, and I feel love for her; she is my friend. She complains a lot without seeing me.”

(Female caregiver)

For many participants, these emotional bonds reinforced their commitment to caregiving and helped sustain them through the challenges of caring for a loved one living with ADRD.

### Preferences of the person living with ADRD

Several participants reported that the preferences of the person living with dementia also influenced caregiver selection. Families respected the wishes of the care recipient regarding who should provide care, recognising that familiarity and trust contributed to the person’s comfort and wellbeing.

As one participant simply stated:

“It was the patient’s decision.”

(Female caregiver)

Overall, caregiver selection reflected an interplay of family relationships, emotional attachment, and respect for the preferences of the person living with ADRD. These factors collectively shaped who assumed the primary caregiving role within the family.

### Theme 2: Multidimensional struggles of caregiving

Participants described caregiving as a demanding and often overwhelming experience that affected nearly every aspect of their lives. The challenges extended beyond caring for the person living with ADRD and included emotional distress, physical exhaustion, financial hardship, disrupted social and occupational lives, and uncertainty about the future. These experiences were interconnected and frequently compounded one another, creating a substantial caregiving burden. Six sub-themes emerged: financial and physical challenges, emotional burden, behavioural challenges, personal sacrifices, fear and uncertainty, and support and coping.

### Financial and physical challenges

Financial strain was one of the most commonly reported challenges. Participants described spending substantial amounts of money on medical care, food, transport, and other daily necessities, often with little or no financial assistance from relatives.

One caregiver explained:

“I spend much money on her. All her needs demand lots of money. My relatives don’t support me often because they think I want to eat their money.”

(Male caregiver)

Another participant reflected on the long-term financial consequences of caregiving:

“When my dad got sick, I took on the responsibility of taking care of him thinking this was going to be temporary and he would get back on his feet soon. However, he never got better. It is now seven years and all my savings have been depleted. I can no longer take care of him and provide the care and support he needs, yet none of my relatives is willing to help.”

(Male caregiver)

Physical caregiving was equally demanding. Participants described assisting with mobility, lifting patients, helping with toileting, cleaning after episodes of incontinence, and managing recurrent falls.

One caregiver shared:

“The patient falls most times and gets wounds on the whole body.”

(Female caregiver)

Another explained:

“I have faced problems taking the patient to the toilet. She is heavy. Many times she just poops on herself and I have to wash her most days. I have even lost my appetite for food.”

(Female caregiver)

For many participants, these physical demands became increasingly difficult as the condition progressed.

### Emotional burden

Participants described considerable emotional distress while witnessing the progressive decline of their loved ones. Many struggled with sadness, helplessness, frustration, and grief as they watched formerly independent family members become increasingly dependent.

One participant reflected:

“I feel bad seeing that he used to care for us but now he needs us to care for him. He was such a strong and hard-working man.”

(Male caregiver)

Others described feelings of helplessness when they were unable to provide the care they believed their relatives deserved.

“I wish I could afford it. There are times when he needs to go to the hospital and I can’t help.”

(Male caregiver)

Several participants also acknowledged experiencing intrusive thoughts, persistent worry, and emotional exhaustion.

One caregiver explained:

“I usually get bad thoughts, sadness and fear of death. I worry all the time that one day he might go away from home and by the time they call me it will be too late. Honestly, I don’t want him to die.”

(Female caregiver)

### Behavioural challenges

Behavioural symptoms associated with dementia were another major source of distress. Participants described managing wandering, irritability, aggression, food refusal, and verbal abuse, all of which increased the complexity of caregiving.

One participant reported:

“Sometimes he disappears and you have to look for him. Sometimes it takes days to find him. And when you do, he’s smelling of alcohol.”

(Female caregiver)

Another caregiver described how behavioural changes affected family interactions.

“My dad gets irritable when he doesn’t get the food he wants. He abuses me with all the obscene words that you can imagine. At times I get very annoyed and abuse him as well, but then I remember he is sick and will not even remember what we just quarrelled about.”

(Male caregiver)

Food refusal was also frequently reported.

“Most times she refuses to eat the food that I have prepared, even when I try milk she does not want it. This usually happens when, for no particular reason, she gets annoyed.”

(Female caregiver)

These behavioural symptoms often left caregivers feeling frustrated, emotionally drained, and uncertain about how best to respond.

### Personal sacrifices

Providing care frequently required participants to sacrifice important aspects of their own lives. Several caregivers described leaving employment, interrupting education, or moving away from their own families to provide full-time care.

One participant explained:

“I cannot handle work, family and taking care of her. I left my job and my husband’s home to come and take care of her.”

(Female caregiver)

Another reflected on abandoning her education.

“I dropped out of school to take care of Grandma. She was the one paying my tuition. By that time I was in my second year at university. It is now five years and all my relatives feel I should be the one to take care of her. They don’t even give me any upkeep money for myself, yet I also have needs.”

(Female caregiver)

These accounts illustrate the significant personal, educational, occupational, and social sacrifices associated with long-term caregiving.

### Fear and uncertainty

Participants also described living with constant uncertainty about the future. Many worried about disease progression, their own ability to continue providing care, and what would happen if the person living with ADRD died.

One caregiver shared:

“If she dies at the moment, what do I do? She is the only relative that I have.”

(Female caregiver)

Others expressed sadness related to multiple health conditions experienced by their loved ones.

“I feel sad because she was amputated. This woman has suffered a lot. On top of all the problems she has, including not remembering any of us, both her legs were cut off.”

(Female caregiver)

These fears often coexisted with uncertainty regarding the future and feelings of emotional vulnerability.

### Support and coping

Despite the numerous challenges, some participants described drawing strength from family, friends, neighbours, and religious faith. Emotional encouragement, financial assistance, and practical support helped caregivers cope with the demands of caregiving.

However, support was inconsistent. While some caregivers felt well supported, many described providing care largely on their own, with limited involvement from other family members.

Overall, participants portrayed caregiving as a complex and demanding responsibility that affected their emotional wellbeing, physical health, financial stability, education, employment, and social relationships. These multidimensional challenges frequently occurred simultaneously and evolved as dementia progressed.

### Theme 3: Benefits of caregiving

Although caregiving was widely described as physically, emotionally, and financially demanding, participants also reflected on positive experiences that emerged from caring for their family members. These experiences included acquiring new knowledge and skills, developing greater empathy, receiving professional guidance, appreciating life differently, experiencing support from others, and finding fulfilment through reciprocity. Together, these experiences helped many caregivers derive meaning from their caregiving role despite the challenges they faced.

### Knowledge and skills acquisition

Participants reported that caring for a person living with ADRD increased their knowledge of dementia and the healthcare system. Many became familiar with available health services and developed confidence in navigating medical care for their relatives.

One caregiver explained:

“Ever since I started caring for him, it has helped me know Mulago and also the doctors who work there. So when I need some medical help, I call them and I get immediate guidance.”

(Female caregiver)

For many participants, these experiences improved their confidence in caring for their relatives and strengthened their ability to seek appropriate healthcare when needed.

### Empathy and personal growth

Caregiving also fostered greater patience, compassion, and understanding. Participants described learning to respond more calmly to behavioural changes and recognising that many difficult behaviours were consequences of the illness rather than intentional actions.

One caregiver reflected:

“I don’t get annoyed because it’s not her. She needs love in order to keep going. I have even learned to be tolerant and patient. It has been a long journey.”

(Female caregiver)

These experiences contributed to personal growth and a deeper appreciation of the emotional needs of persons living with dementia.

### Professional guidance and preparedness

Several participants highlighted the importance of advice received from healthcare professionals. Guidance from clinicians helped them understand disease progression, anticipate future challenges, and feel better prepared to provide care.

One participant shared:

“Doctors help. I was connected to doctors in Mulago who would tell me what to expect and how to prepare myself for any changes in my patient.”

(Female caregiver)

Access to professional guidance increased caregivers’ confidence and reduced some of the uncertainty associated with caring for a person living with ADRD.

### Life appreciation and social awareness

The caregiving experience also prompted participants to reflect on life, relationships, and the realities of illness. Some described gaining a deeper appreciation for life, while others became more aware of the social isolation that can accompany chronic illness.

As one caregiver explained:

“I have learnt to appreciate life and understand that your people can forget about you when you are sick.”

(Female caregiver)

These reflections often extended beyond caregiving itself and influenced participants’ broader perspectives on family and social relationships.

### Support from family and community

Participants acknowledged the important role played by friends, neighbours, faith communities, and other social networks in helping them cope with caregiving responsibilities. Support included emotional encouragement, financial assistance, material support, and spiritual care.

One participant explained:

“Friends and neighbours have been financially, spiritually and emotionally helpful and have even provided food and clothing.”

(Female caregiver)

Although support was not available to everyone, caregivers who received assistance described it as an important source of encouragement during difficult periods.

### Reciprocity and fulfilment

For many participants, caregiving represented an opportunity to give back to family members who had previously cared for them. Rather than viewing caregiving solely as an obligation, they described it as an expression of gratitude and appreciation.

One caregiver reflected:

“I have hope that I have given back to her for having raised me.”

(Female caregiver)

These accounts suggest that, despite considerable hardship, caregiving could also foster a sense of fulfilment, purpose, and personal meaning.

Overall, participants described caregiving as a complex experience characterised by both substantial challenges and meaningful personal rewards. While caregiving imposed considerable emotional, physical, social, and financial demands, many caregivers also reported personal growth, stronger relationships, increased knowledge, and a sense of fulfilment through caring for a loved one living with ADRD.

## Discussion

Caregiving responsibilities in Uganda emerged from relational and reciprocal family dynamics rather than formal assignment, highlighting the cultural roots of caregiving. These relational foundations influenced who assumed caregiving responsibilities, how those responsibilities were sustained over time, and how caregivers interpreted both the hardships and rewards of caring for a family member living with dementia. The caregiving role was taxing across emotional, physical, and financial domains, yet participants also described it as a source of empathy and fulfilment. These accounts reveal caregiving as both hardship and resilience, offering a richer perspective than burden-focused studies alone.

Similar caregiving dynamics have been reported in Nigeria, where families navigate dementia care through biomedical, faith-based, and traditional systems [21], and in South Africa, where caregivers in low-income townships balance significant hardship with protective factors and resilience [22]. These regional parallels highlight that while caregiving is demanding, it also fosters reciprocity and resilience across African contexts, underscoring the need for culturally tailored interventions that recognize both hardship and benefit.

### Caregiving as a relational and moral responsibility

A central finding was that caregiver selection was rarely based on a formal assessment of availability, resources, or caregiving skills. Instead, caregiving responsibilities emerged from existing family relationships. Participants became caregivers because they were considered close to the person living with dementia, had previously received care or support from them, or were specifically preferred by the care recipient. Caregiver selection was therefore closely linked to shared history, emotional familiarity, and the perceived responsibility to care for a family member who had previously cared for others.

This finding extends beyond the observation that most dementia care is provided by family members. It suggests that, within this setting, caregiving was understood as a continuation of an established relationship rather than the beginning of a new role. The relationship between caregiver and care recipient preceded the illness and shaped who was considered most suitable to provide care. Similar findings have been reported in other cultural contexts, where caregiving is motivated by reciprocal love, filial responsibility, and an expectation that support received earlier in life should be returned during illness or old age [15]. Research from sub-Saharan Africa has also shown that family commitment, affection, social expectations, and the absence of alternative caregivers influence decisions to provide dementia care [16].

However, describing caregiving as a family responsibility should not imply that the role was freely chosen or equally shared. Although participants frequently expressed love and gratitude, some accounts suggested that caregiving responsibility became concentrated in one family member. Closeness to the care recipient could make a person the preferred caregiver, but it could also make it more difficult for them to decline or redistribute the role. In this way, relational closeness functioned both as a source of commitment and as a pathway through which long-term responsibility was assigned.

The expressed preferences of persons living with ADRD also influenced caregiver selection. This finding is important because it shows that care recipients were not entirely passive within family decision-making. Familiarity and trust may be particularly valuable for individuals experiencing cognitive decline, behavioural changes, or difficulty adapting to unfamiliar people. Respecting these preferences may support dignity and emotional security. Nevertheless, exclusive dependence on one preferred caregiver may also increase strain when the caregiver has limited financial resources, competing family responsibilities, or poor health. Care planning should therefore respect the care recipient’s preferences while also considering the caregiver’s capacity and the availability of additional support.

### Multidimensional burden and cumulative sacrifice

The challenges reported by participants were emotional, physical, financial, occupational, educational, and social. These difficulties did not occur independently. Rather, they accumulated and reinforced one another. For example, the physical demands of lifting, toileting, cleaning, and supervising a person at risk of wandering were accompanied by emotional distress and reduced opportunities for paid employment. Financial limitations then restricted access to medical care, hygiene supplies, transport, and additional caregiving assistance, further increasing the caregiver’s workload and sense of helplessness.

These findings are consistent with evidence showing that dementia caregiving is associated with psychological distress, anxiety, depression, and reduced quality of life [7–9]. They are also consistent with Ugandan studies reporting substantial caregiving burden and mental health difficulties among family caregivers of persons living with dementia [17,18]. In our previous study in Wakiso District, psychological distress and depression were each reported by 64.4% of caregivers, demonstrating that the emotional difficulties described in the present interviews form part of a broader and measurable mental health burden among caregivers in this setting [18].

The present study adds depth to this evidence by showing how caregiver distress developed within everyday care. Participants described witnessing the decline of relatives who had previously been strong, independent, and responsible for supporting the family. This reversal of roles was painful because caregivers were not only responding to current care needs but also grieving the gradual loss of the person they had known. Feelings of sadness, fear, helplessness, and uncertainty were therefore closely tied to changes in the care recipient’s identity, abilities, and place within the family.

Behavioural symptoms further intensified caregiver distress. Wandering, irritability, verbal aggression, food refusal, and alcohol use created safety concerns and strained relationships. Behavioural and psychological symptoms are common among persons living with dementia and can cause substantial caregiver distress. Evidence from Uganda has similarly shown a high occurrence of behavioural symptoms, with some symptoms producing severe distress among caregivers [19]. In the present study, caregivers understood that some behaviours were related to illness, but this understanding did not always prevent frustration or reactive conflict. One caregiver, for example, described responding verbally to abuse before remembering that the care recipient was unwell and unlikely to recall the exchange. Such accounts should not be reduced to individual caregiver failure. They point to the risks that arise when caregivers are expected to manage complex behavioural symptoms without adequate information, practical skills, respite, or professional support.

The physical and hygiene-related demands of care were particularly striking. Participants described lifting heavy care recipients, managing falls, cleaning faecal incontinence, and providing intimate care without gloves or other basic supplies. These accounts demonstrate how material deprivation directly shaped both caregiver wellbeing and the quality and safety of care. Expressions of disgust, loss of appetite, and fear of illness should therefore be understood within the context of repeated exposure to difficult care tasks without appropriate equipment or assistance. Caregiver education alone will be insufficient where families cannot afford gloves, continence products, transport, medical treatment, or additional help.

Financial strain was also central to the caregiving experience. Some participants depleted their savings, left employment, or became unable to meet the care recipient’s health needs. Others felt abandoned or mistrusted by relatives when requesting financial assistance. These findings show that the economic consequences of dementia extend beyond direct medical expenses. They include lost income, interrupted education, reduced productivity, and long-term changes in caregivers’ life opportunities. Informal care accounts for a substantial share of dementia-related costs in low- and middle-income countries, where limited formal services shift much of the responsibility to households and families.

The personal sacrifices described by participants were therefore not minor adjustments. Leaving employment, moving away from a spouse, or withdrawing from university altered caregivers’ economic security, family relationships, and future prospects. These consequences may be especially severe for younger caregivers who interrupt education or career development during important stages of their lives. The findings indicate that caregiver burden should not be assessed only in terms of current stress or hours of care. It should also include lost opportunities and the long-term social and economic consequences of assuming an intensive caregiving role.

### Positive experiences, meaning, and reciprocity

Despite the substantial challenges, caregivers did not describe their experiences entirely in negative terms. Participants reported gaining practical knowledge, learning how to navigate health services, becoming more patient and empathetic, and receiving support from healthcare professionals and community members. They also described caregiving as an opportunity to give back to relatives who had previously raised or supported them.

These findings demonstrate the importance of recognising both the burdens and the positive meanings of caregiving. Positive experiences did not remove financial strain, exhaustion, or distress. Rather, they existed alongside these challenges. A caregiver could feel overwhelmed by the demands of care while simultaneously experiencing love, gratitude, personal growth, or fulfilment. Presenting these experiences together provides a more complete understanding of caregiving than an exclusive focus on burden.

Reciprocity was particularly important. Participants frequently interpreted caregiving in relation to earlier support received from the care recipient. Caring for a parent or grandparent was described as a way of returning care, expressing gratitude, and honouring a longstanding relationship. This interpretation may help caregivers sustain commitment during difficult periods by connecting daily caregiving tasks to a broader moral and relational purpose. Similar work has identified reciprocal love and filial responsibility as important moral frameworks through which family members understand dementia caregiving [15].

However, the positive meaning attached to reciprocity should be interpreted carefully. Gratitude and fulfilment may strengthen resilience, but they can also coexist with unrecognised exhaustion or make caregivers feel unable to ask for help. A caregiver who understands care as repayment may feel that sharing responsibility or expressing frustration represents a failure of love or duty. Interventions should therefore acknowledge and build upon caregivers’ values without romanticising sacrifice or treating family obligation as a substitute for practical support.

Professional guidance was another important positive experience. Caregivers valued being connected to clinicians who explained what to expect and advised them on managing changes in the care recipient. This suggests that even relatively simple access to reliable information may reduce uncertainty and strengthen caregiving confidence. The World Health Organization’s iSupport programme similarly combines dementia education, caregiver self-care, everyday care skills, and strategies for responding to behavioural changes. It is designed for adaptation to local contexts and can be delivered in formats suitable for settings with limited internet access.

Support from friends, neighbours, and faith communities also provided emotional, spiritual, financial, and material assistance. In a context where formal dementia services remain limited, these community relationships may represent an important source of resilience. However, support was unevenly distributed, and several caregivers described being left to provide care with little assistance from relatives. Community support should therefore complement, rather than replace, formal health and social support.

### Implications for caregiver support and dementia services

The findings have several implications for dementia care in Uganda and similar low-resource settings. First, caregiver support should begin with an assessment of both the care recipient’s needs and the caregiver’s capacity. Health workers should identify who is providing most of the care, whether that person has chosen the role, the tasks they are performing, and the effect of caregiving on their physical health, mental wellbeing, education, employment, and family life.

Second, caregivers require practical training that responds to the problems they face at home. Priority areas include understanding dementia, managing behavioural changes, preventing and responding to wandering and falls, assisting with feeding and personal care, managing incontinence, communicating calmly during episodes of agitation, and recognising when professional assessment is required. Training should also include caregiver self-care, problem-solving, and strategies for mobilising family and community support. WHO recommends education, training, respite, psychological support, and financial assistance as part of a broader response to caregiver wellbeing [20].

Third, caregiver interventions should recognise the relational foundations of care. Programmes that frame caregiving only as a source of burden may not fully resonate with caregivers who understand their role through love, gratitude, duty, and reciprocity. Culturally adapted interventions should validate these motivations while also reinforcing that accepting help, sharing responsibilities, and attending to one’s own health are compatible with being a committed caregiver.

Fourth, support should extend beyond education. The financial and material challenges identified in this study require social and policy responses, including improved access to affordable health services, basic caregiving supplies, respite options, income protection, and mechanisms for linking families to community resources. Family meetings may also help redistribute tasks and clarify that responsibility for dementia care should not fall entirely on the family member who is emotionally closest or most readily available.

Community health workers or Village Health Teams may be well placed to provide basic dementia education, monitor caregiver wellbeing, reinforce practical skills, and connect families with health facilities. Peer-support groups may further reduce isolation by enabling caregivers to share experiences and locally developed solutions. These approaches should be evaluated for feasibility, acceptability, and effectiveness within Ugandan communities rather than transferred directly from settings with different family and health-system structures.

### Strengths and limitations

A strength of this study is its detailed account of caregiving experiences across urban and rural communities in Wakiso District. The inclusion of caregivers with different relationships to care recipients allowed the study to capture varied pathways into caregiving and a broad range of emotional, physical, and socioeconomic experiences. Conducting interviews in Luganda supported participants to describe sensitive and complex experiences in their preferred language. The use of the Mental Models Framework also helped examine how caregivers understood and gave meaning to their responsibilities rather than focusing only on the tasks they performed.

The findings should nevertheless be considered in light of several limitations. The study included 14 caregivers from two communities in one district, and the findings may not represent all dementia caregiving experiences in Uganda. Participants were recruited from a previous study cohort, which may have increased their familiarity with dementia research and health services compared with caregivers who have not previously participated in research. Most participants were women, although this reflects the gendered distribution of informal caregiving reported more broadly. The study also relied on caregivers’ accounts and did not include the perspectives of persons living with ADRD, other family members, or healthcare providers. Interviews were translated from Luganda into English, and some cultural meaning may have been reduced despite transcript checking and supervision. Finally, because the findings were generated through qualitative interviews, they explain how participants understood their experiences but cannot determine the prevalence or relative importance of each challenge across the wider caregiver population.

Future studies should examine how caregiving roles change over time, how families negotiate or redistribute responsibilities, and how caregiving affects education, employment, health, and household finances over the longer term. Research should also evaluate culturally adapted, community-supported interventions that combine dementia education, behavioural management skills, caregiver mental health support, peer connection, and practical assistance.

### Implications for practice

These findings highlight the urgent need for culturally tailored caregiver support in Uganda and similar low-resource settings. Interventions should move beyond burden reduction alone to incorporate training in practical skills, psychosocial support, and community-based resources that recognize caregiving as both hardship and resilience. By acknowledging the dual nature of caregiving, programs can strengthen caregiver wellbeing, foster reciprocity within families, and improve the quality of dementia care across sub-Saharan Africa.

## Conclusion

Caregiving for persons living with ADRD in Uganda was grounded in longstanding family relationships, emotional closeness, and a sense of reciprocal responsibility. These relational foundations strengthened caregivers’ commitment but also concentrated responsibility in individuals who often had limited preparation, resources, or support. As dementia progressed, caregiving affected emotional wellbeing, physical health, finances, education, employment, and family life. At the same time, caregivers described personal growth, empathy, and fulfilment through giving back to relatives who had previously cared for them.

Support for dementia caregivers in low-resource settings must therefore move beyond a narrow focus on burden. Effective interventions should recognise the cultural and relational meanings that sustain caregiving while addressing the practical conditions that make it difficult. Culturally adapted training, psychosocial support, and community-based resources are essential to protect caregiver wellbeing and strengthen the sustainability of home-based dementia care.

## Supplementary Material

Supplementary Files

This is a list of supplementary files associated with this preprint. Click to download.

- BMCSupplementaryFile1InterviewGuide.docx

## Figures and Tables

**Table 1:** Sociodemographic characteristics of caregivers (N = 14)

Characteristic	n	%
**Gender**		
Male	5	35.7
Female	9	64.3
Highest level of education		
Primary	3	21.4
Secondary	7	50.0
Tertiary	4	28.6
Main source of income		
None	8	57.1
Gardening	2	14.3
Business	3	21.4
Professional employment	1	7.1
Health comorbidities		
Diabetes	1	7.1
Hypertension	2	14.3
HIV	1	7.1
None	10	71.4
Relationship to care recipient		
Spouse	3	21.4
Adult child	4	28.6
Grandchild	3	21.4
Sibling	2	14.3
In-law	1	7.1
Paid caregiver	1	7.1

**Table 2:** Summary of themes and subthemes

Theme	Subthemes
Relational foundations of caregiving	Familial relationships and responsibility Emotional connections Preferences of the person living with ADRD
Multidimensional struggles of caregiving	Financial and physical challenges Emotional burden Behavioural challenges Personal sacrifices Fear and uncertainty Support and coping
Benefits of caregiving	Knowledge and skills acquisition Empathy and personal growth Professional guidance and preparedness Life appreciation and social awareness Support from family and community Reciprocity and fulfilment

## Data Availability

The datasets used and analyzed during the current study are available from the corresponding author upon reasonable request.

## Abbreviations

ADRD: Alzheimer’s disease and related dementias
HDREC: Higher Degrees Research and Ethics Committee
UNCST: Uganda National Council for Science and Technology
OD: Office of the Director, National Institutes of Health
NIDCR: National Institute of Dental & Craniofacial Research
NINDS: National Institute of Neurological Disorders and Stroke
NHLBI: National Heart, Lung, and Blood Institute
FIC: Fogarty International Center
NIMHD: National Institute on Minority Health and Health Disparities
